# Pleiotropic Regulator GssR Positively Regulates Autotrophic Growth of Gas-Fermenting *Clostridium ljungdahlii*

**DOI:** 10.3390/microorganisms11081968

**Published:** 2023-07-31

**Authors:** Huan Zhang, Can Zhang, Xiaoqun Nie, Yuwei Wu, Chen Yang, Weihong Jiang, Yang Gu

**Affiliations:** 1 CAS-Key Laboratory of Synthetic Biology, CAS Center for Excellence in Molecular Plant Sciences, Shanghai Institute of Plant Physiology and Ecology, Chinese Academy of Sciences, 300 Fenglin Road, Shanghai 200032, China; 2 University of Chinese Academy of Sciences, Beijing 100049, China

**Keywords:** transcription factors, GssR, metabolic regulation, *C. ljungdahlii*, gas fermentation

## Abstract

*Clostridium ljungdahlii* is a representative autotrophic acetogen capable of producing multiple chemicals from one-carbon gases (CO_2_/CO). The metabolic and regulatory networks of this carbon-fixing bacterium are interesting, but still remain minimally explored. Here, based on bioinformatics analysis followed by functional screening, we identified a RpiR family transcription factor (TF) that can regulate the autotrophic growth and carbon fixation of *C. ljungdahlii*. After deletion of the corresponding gene, the resulting mutant strain exhibited significantly impaired growth in gas fermentation, thus reducing the production of acetic acid and ethanol. In contrast, the overexpression of this TF gene could promote cell growth, indicating a positive regulatory effect of this TF in *C. ljungdahlii*. Thus, we named the TF as GssR (growth and solvent synthesis regulator). Through the following comparative transcriptomic analysis and biochemical verification, we discovered three important genes (encoding pyruvate carboxylase, carbon hunger protein CstA, and a BlaI family transcription factor) that were directly regulated by GssR. Furthermore, an upstream regulator, BirA, that could directly bind to *gssR* was found; thus, these two regulators may form a cascade regulation and jointly affect the physiology and metabolism of *C. ljungdahlii*. These findings substantively expand our understanding on the metabolic regulation of carbon fixation in gas-fermenting *Clostridium* species.

## 1. Introduction

One-carbon (C1) gases (e.g., CO_2_ and CO) are attractive substrates for microbial fermentation since they are abundant in industrial syngas or waste gases [1]. The biological conversion of C1 gases to various value-added products, including bulk chemicals and biofuels, has attracted widespread attention recently because it provides an alternative to the traditional biomanufacturing process, which mainly depends on starch and sugar materials [2,3]. Autotrophic *Clostridium* species can assimilate CO_2_ and CO via the Wood–Ljungdahl pathway (WLP) to synthesize multiple native chemicals, such as acetic acid, ethanol, butyric acid, butanol, and 2,3-butanediol [4,5,6,7], thereby representing an important C1 chassis. Of note, large-scale ethanol production using steel mill off-gases has been achieved by using some gas-fermenting *Clostridium* species, such as *Clostridium autoethanogenum* and *C. ljungdahlii* [8], showcasing the great application prospect of these bacteria.

*C. ljungdahlii* is one of the representative species of gas-fermenting *Clostridium*. It was originally isolated from chicken yard waste [9] and can use both CO_2_/CO and sugars as carbon sources to support growth [4]. The previous studies revealed that significant differences occurred in the genome-wide transcription profiles of *C. ljungdahlii* grown on sugars and CO_2_/CO [10], and furthermore, a large number of differentially expressed genes were found to be potentially associated with C1 gas metabolism [11]. These findings indicate that *C. ljungdahlii* has unique regulatory mechanisms to adapt to gas fermentation.

To better understand the physiology and metabolism of *C. ljungdahlii* in gas fermentation, discovery of crucial regulators as well as dissection of the underlying regulatory mechanism are necessary, but till now, this aspect has remained minimally explored. To our knowledge, the reported regulatory factors with detailed analysis in *C. ljungdahlii* include CcpA, Rex, and BirA. Among them, CcpA could directly regulate the WLP genes and thus, alleviation of CcpA’s inhibition on these genes could improve CO_2_ utilization of *C. ljungdahlii* [12]. The transcription factor Rex was found to regulate crucial genes responsible for acid re-assimilation and ethanol formation in *C. ljungdahlii* [13]; BirA was proven to serve as both a transcriptional regulator and biotin ligase in *C. ljungdahlii*, and the inactivation of *birA* severely impaired the cell growth on gas [14]. All these findings indicate the importance of TFs for *C. ljungdahlii*, thereby strongly supporting further investigation regarding their regulatory mechanisms. This will not only deepen our understanding on this autotrophic bacterium but also guide rational strain modifications.

In this study, based on the comparative transcriptomic data and experimental verification, we identified a novel TF, GssR, in *C. ljungdahlii*. This regulator was found to be involved in the regulation of autotrophic growth and product synthesis of this bacterium. Through further investigation, we discovered multiple genes that are directly or indirectly controlled by GssR. An upstream regulator BirA that can directly bind to the *gssR* gene was also identified, thereby generating a potential complex regulatory circuit that employs both GssR and BirA. These findings effectively expand the understanding on the physiology and metabolism of autotrophic gas-fermenting *Clostridium* species.

## 2. Materials and Methods

### 2.1. Reagents, Bacteria, and Media

All the strains used in this study are listed in Supplementary Table S1.

The *E. coil* DH5α and its derived strains were cultured in the LB (lysogeny broth) medium [15]. The *C. ljungdahlii* DSM 13528 and its derived strains were cultured in the YTF (yeast extract–tryptone–fructose) medium [16] for inoculum preparation and a modified ATCC 1754 medium [17] for gas fermentation (CO−CO_2_−H_2_−N_2_; 56%/20%/9%/15%; pressurized to 0.2 MPa). All the manipulation of *C. ljungdahlii* strains was performed in an anaerobic chamber (Whitley A35 workstation, don Whitley Scientific Limited, Bingley, West Yorkshire, UK) at 37 °C. Chloramphenicol (12.5 μg/mL) and thiamphenicol (5 μg/mL) were added into the media when needed.

The *Clostridium acetobutylicum* and *Clostridium beijerinckii* strains were cultured in the CGM medium [18] for inoculum preparation and the P2 medium [19] for fermentation. All the manipulations of *C. acetobutylicum* and *C. beijerinckii* strains were performed in an anaerobic chamber (Thermo Fisher Scientific Inc., Waltham, MA, USA) at 37 °C. Erythromycin (10 μg/mL) was added into the media when needed.

KOD plus and KOD FX DNA polymerases (Toyobo, Osaka, Japan) were used for PCR amplification. All the primers were synthesized by Biosune (Biosune, Shanghai, China). The enzymes were purchased from Thermo Fisher Scientific (Thermo Fisher Scientific, Vilnius, Lithuania). DNA fragments and linear plasmids were assembled using a ClonExpress II One Step Cloning Kit (Vazyme Biotech Co., Ltd., Nanjing, China). Plasmid isolation and DNA purification were performed using the kits (Axygen Biotechnology Company Limited, Hangzhou, China) according to the manufacturer’s protocols.

### 2.2. Plasmid Construction

The plasmids and primers used in this study are listed in Supplementary Tables S2 and S3, respectively.

The pMTLcas*-gssR* plasmid for the deletion of *gssR* was constructed as follows. Firstly, a sgRNA fragment targeting the 20 nt target spacer of the *gssR* gene (designed on https://benchling.com, assessed on 12 June 2019) was obtained through PCR amplification using the pMTLcas plasmid as the template and the primers *gssR* gRNA-for/*gssR* gRNA-rev. Next, the two homologous arms (HAs) that flank the coding region of *gssR* were obtained through PCR amplification using the *C. ljungdahlii* genomic DNA as the template and the primers *gssR*-UpArm-for/*gssR*-UpArm-rev and *gssR*-DownArm-for/*gssR*-DownArm-rev. The sgRNA and the two HAs fragments were then linked together via overlapping PCR using the primers *gssR*-gRNA-for/*gssR*-DownArm-rev, yielding a DNA fragment sgRNA-HA. Finally, the pMTLcas-*pta* [17] plasmid was digested with both *Sal*I and *Xho*I, and the resulting linear pMTLcas-*pta* vector was assembled with the sgRNA-HA fragment by using the ClonExpress II One Step Cloning Kit (VazymeBiotech, Nanjing, China), yielding the pMTLcas*-gssR* plasmid.

The pMTL83151-P*_gssR_*-*gssR* plasmid for the overexpression of *gssR* was constructed as follows. The pMTL83151 [20] plasmid was treated with both *Nde*I and *Hind*III, yielding a linear vector. The DNA fragment P*_gssR_*-*gssR* was obtained through PCR amplification using the *C. ljungdahlii* genomic DNA as the template and the primers P*_gssR_*-for/*gssR*-rev. Next, the linear pMTL83151 vector and the P*_gssR_*-*gssR* fragment were assembled using the ClonExpress II One Step Cloning Kit, yielding the plasmid pMTL83151-P*_gssR_*-*gssR*. The other plasmids for gene overexpression in *C. ljungdahlii* were constructed following the same steps.

The pZG-ddFncas12a-37350 plasmid for the transcriptional repression of CLJU_c37350 via CRISPR interference (CRISPRi) was constructed as follows. In brief, a double-strand DNA (harboring the crRNA spacer and repeat sequences) (designed on https://benchling.com, 7 January 2020) was obtained through PCR amplification using the primers 37350-crRNA-for/37350-crRNA-rev. Next, this DNA fragment was adopted as the template for further PCR amplification using the primers spacer-for/spacer-rev, yielding a new DNA fragment that contained the crRNA sequence and a terminator. Finally, this DNA fragment was assembled with the linear pZG-ddFncas12a plasmid (*BamH*I/*Nco*I digestion) using the ClonExpress II One Step Cloning Kit, yielding the pZG-ddFncas12a-37350 plasmid. The other plasmids for CRISPRi were constructed following the same steps except using different crRNAs.

The pET28a-*gssR* plasmid for protein expression in *E. coil* DH5α was constructed as follows. Firstly, the *gssR* gene was obtained through PCR amplification by using the *C. ljungdahlii* genomic DNA as the template and the primers pET28a-*gssR*-F/pET28a-*gssR*-R. Next, the pET28a plasmid was digested with both *Nde*I and *Xho*I. The resulting linear pET28a plasmid was assembled with the DNA fragment of *gssR* by using the ClonExpress II One Step Cloning Kit (VazymeBiotech, Nanjing, China), yielding the pET28a-*gssR* plasmid.

The pWJ1-CAC1850 plasmid for the disruption of the CAC1850 gene was constructed as follows. In brief, A 350-bp DNA fragment was first obtained through PCR amplification by using the following primers: the EBS universal primer, CAC1850-381,382s-IBS, CAC1850-381,382s-EBS1d, and CAC1850-381,382s-EBS2. Amplification was performed according to the protocol of the Targetron gene knockout system kit (Sigma-Aldrich, St. Louis, MO, USA). Next, the plasmid pWJ1 [21] was digested with *Xho*I and *BsrG*I. Finally, this 350-bp DNA fragment was assembled with the linear pWJ1 vector, yielding the plasmid pWJ1-CAC1850.

### 2.3. Analytical Methods

Cell growth was determined based on the absorbance of the culture at *A*_600_ (OD_600_) using a spectrophotometer (DU730, Beckman Coulter, Brea, CA, USA). The concentrations of products (acetate and ethanol) were measured according to the methods described previously [17]. Briefly, samples were taken at appropriate time intervals and then harvested using centrifugation (7000× *g* for 10 min at 4 °C). The concentrations of acetate and ethanol in the supernatant were determined using a 7890A gas chromatograph (Agilent, Wilmington, DE, USA) equipped with a capillary column (EC-Wax, Alltech, Lexington, KY, USA) and a flame ionization detector (Agilent, Wilmington, DE, USA).

### 2.4. Comparative Transcriptomic Analysis by RNA-seq

The *gssR*-disrupted and the wild-type *C. ljungdahlii* strains were grown in the modified ATCC medium 1754 (100 mL) with a headspace of syngas (CO−CO_2_−H_2_−N_2_; 56%/20%/9%/15%; pressurized to 0.2 MPa). Samples for RNA-seq were taken when the optical density (OD_600_) of grown cells reached ~1.0 and then subjected to centrifugation (5000× *g*, 4 °C for 10 min). Cell pellets were collected and frozen immediately in liquid nitrogen before RNA extraction. Total RNA was extracted using a kit (cat#cw0581; CWBIO, Beijing, China) according to the manufacturer’s protocol and then treated with DNase I (TaKaRa, Kyoto, Japan) to eliminate DNA contained in RNA samples. The concentration of total RNA was determined using a NanoDrop spectrophotometer (Thermo Fisher Scientific Inc., Waltham, MA, USA). High-quality RNA samples were used to construct sequencing libraries. The preparation of the RNA-seq transcriptome library and data analysis were performed the same as described previously [22].

### 2.5. Real-Time qRT-PCR

The *C. ljungdahlii* strains were grown anaerobically at 37 °C. Cells were harvested when the optical density (OD_600_) reached ~1.0. The total RNA was isolated using a kit (cat#cw0581; CWBIO, Beijing, China) and then treated using DNase I (TaKaRa, Kyoto, Japan) to eliminate DNA contained in RNA samples. The concentration of extracted RNA was determined by using a NanoDrop spectrophotometer (Thermo Fisher Scientific Inc., Waltham, MA, USA). Reverse transcription was performed to produce cDNA by using a PrimeScript RT reagent kit (TaKaRa, Kyoto, Japan). The real-time qPCR was carried out in a CFX Duet real-time PCR system (Bio-Rad, Hercules, CA, USA) following the same steps as described previously [23]. The reaction mixtures (20 μL) contained 1 × iQ SYBR green Supermix (Bio-Rad, Hercules, CA, USA), 0.5 μM of each primer, and the diluted cDNA template (0.625 ng/μL). Here, the *rho* gene (CLJU_c02220) was adopted as the internal control [24].

### 2.6. Overexpression and Purification of His6-Tagged GssR Protein

The pET28a-*gssR* plasmid was transformed into *E. coli* BL21 (DE3) for *gssR* overexpression. Gene expression was induced at 16 °C for 18 h by adding 0.5 mmol/L of isopropyl-β-D-thio-galactoside (IPTG) when cell density (OD_600_) reached 0.8. The grown cells were harvested via centrifugation (5000× *g*, 10 min at 4 °C), washed with a solution (20 mM of Tris-HCl, pH 7.9, 500 mM of KCl, 10% glycerol, and 10 mM of imidazole), and then disrupted with a French press (Constant Systems Limited, Daventry, Northants, UK). Cell debris and membrane fractions were separated from the soluble fraction via centrifugation (10,000× *g*, 60 min at 4 °C). The soluble fraction was loaded onto a Ni Sepharose™ 6 fast-flow agarose (GE Healthcare, Waukesha, WI, USA) column for the purification of His-tagged GssR protein. Protein was eluted by using a buffer (pH 7.9) containing 20 mM of Tris-HCl, 500 mM of KCl, 10% (*v*/*v*) glycerol, and 500 mM of imidazole. The eluent fraction was then transferred to an Amicon Ultra-15 Centrifugal Filter (Milipore, Billerica, MA, USA) and eluted three times by using a buffer (pH 7.9) containing 20 mM of Tris-HCl, 500 mM of KCl, and 10% (*v*/*v*) glycerol. The purified protein was stored at −80 °C.

### 2.7. Electrophoretic Mobility Shift Assay (EMSA)

DNA probes labeled with cyanine 5 (Cy5) were generated through a two-step PCR amplification: first, double-stranded DNA fragments were obtained with PCR amplification using the genomic DNA of *C. ljungdahlii* as the template and specific primer pairs E-fw/E-rev containing a universal primer sequence (5′-AGCCAGTGGCGATAAG-3′) at the 5′ terminus; next, a Cy5-tag was added to the above DNA fragments via PCR amplification using the universal primer (5′-AGCCAGTGGCGATAAG-3′) labeled with Cy5. The resulting Cy5-labeled probes were analyzed using agarose gel electrophoresis, recovered using a PCR purification kit (Axygen Biotechnology Company Limited, Hangzhou, China), and then used for EMSAs.

EMSAs were carried out as follows. In brief, the His-tagged GssR protein was pre-incubated with 0.04 pmol of Cy5-labeled probes in a buffer (20 mM of Tris-HCl, pH 7.9, 5% glycerol, 40 ng/mL bovine serum albumin, 0.25 mM of DTT, 10 mM of MgCl_2_, 20 mM of KCl, and 50 ng/μL fish sperm DNA). The reaction mixture was incubated at 25 °C for 20 min. A 1.5% agarose gel was prepared and pre-run in the 0.5 × TAE buffer at 80 V for 30 min in an ice bath. Then, the reaction mixture was loaded on the agarose gel (20 μL per lane) for electrophoresis (80 V, 90 min). The gel was visualized using a Starion FLA-9000 Scanner (FujiFilm, Tokyo, Japan).

### 2.8. Phylogenetic Tree Construction

Gene annotations were derived from the SEED [25] and KEGG database [26]. Comparative genomic analysis was performed to predict genes with unknown functions by using GenomeExplorer [27]. Multiple protein alignments were carried out using MUSCLE [28] and MAFFT [29]. The phylogenetic tree was constructed via the maximum likelihood method implemented in PhyML [30] and MEGA [31] and further edited with iTOL [32].

### 2.9. Fermentation

The inoculum preparation and anaerobic fermentation of the *C. ljungdahlii* strains were carried out in the YTF medium and the modified ATCC medium 1754, respectively. Thiamphenicol (5 μg/mL) was added into the media when needed. Briefly, 500 μL of frozen stock was added into 5 mL of the liquid YTF medium and then incubated anaerobically at 37 °C for 24 h. When the optical density (OD_600_) of grown cells reached ~1.0, 1.5 mL of cells was transferred into 30 mL of the modified ATCC medium 1754 with a headspace of syngas (CO−CO_2_−H_2_−N_2_; 56%/20%/9%/15%; pressurized to 0.2 MPa) for gas fermentation. The fresh gases were injected into the headspace (again at 0.2 MPa) every 24 h.

The inoculum preparation and anaerobic fermentations of the *C. acetobutylicum* and *C. beijerinckii* strains were carried out in the CGM medium and the P2 medium, respectively. Erythromycin (10 μg/mL) was added to the media when needed. Briefly, 500 μL of frozen stock was added into 5 mL of the liquid CGM medium and then incubated anaerobically at 37 °C for 12 h. When the optical density (OD_600_) of grown cells reached ~1.0, 1.5 mL of cells was transferred into 30 mL of the P2 medium for fermentation.

## 3. Results

### 3.1. Discovery of GssR That Is Capable of Regulating the Autotrophic Growth of C. ljungdahlii

To discover crucial transcription factors (TFs) associated with carbon fixation or product synthesis of *C. ljungdahlii*, we previously predicted over 400 potential TF genes [14] using the pfam database [33]. Based on this, we further screened functional TFs that are associated with autotrophic growth and product formation of *C. ljungdahlii* in gas fermentation via CRISPR-Cas9-based gene deletion [17].

**Figure 1:**
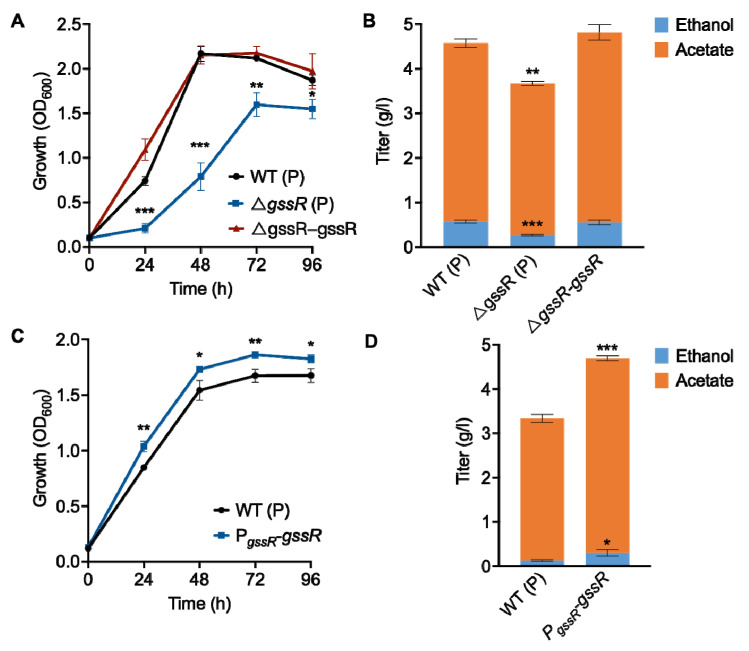
Identification of *gssR* as a crucial regulator in *C. ljungdahlii*. (**A**) The influence of the deletion of *gssR* on strain growth in gas fermentation. WT (P): the wild-type strain containing a blank plasmid. Δ*gssR* (P): the *gssR*-deleted strain containing a blank plasmid. Δ*gssR*-*gssR*: the *gssR*-deleted strain with the re-introduction of *gssR* for expression. (**B**) The influence of the deletion of *gssR* on the production of acetic acid and ethanol in gas fermentation. (**C**) The influence of the overexpression of *gssR* on strain growth in gas fermentation. (**D**) The influence of the overexpression of *gssR* on the production acetic acid and ethanol in gas fermentation. The data are represented as mean ± standard deviation (SD) (*n* = 3). Error bars show SDs. Statistical analysis was performed using a two-tailed Student’s *t*-test. *, *p* < 0.05; **, *p* < 0.01; ***, *p* < 0.001; versus the control (the wild-type strain).

Among the candidates, the deletion of the CLJU_c21350 gene, encoding an RpiR family TF, was found to significantly impair the growth and production of acetic acid and ethanol of *C. ljungdahlii* grown on syngas (CO_2_/CO) (Figure 1A,B). The re-introduction of the CLJU_c21350 gene into the mutant strain completely restored the cell growth and product formation (Figure 1A,B), thereby confirming the causal relationship between this gene and phenotypic outcomes. Here, we named this regulator GssR (growth and solvent synthesis regulator). Furthermore, *gssR* was overexpressed in *C. ljungdahlii* to examine phenotypic changes; as expected, the overexpression of this gene enhanced the growth and production of acetic acid and ethanol of *C. ljungdahlii* in gas fermentation (Figure 1C,D). Therefore, these results demonstrate that GssR plays a crucial regulatory role in *C. ljungdahlii*.

### 3.2. Comparative Transcriptomic Analysis Revealed Potential Genes Subjected to GssR’s Regulation

**Figure 2:**
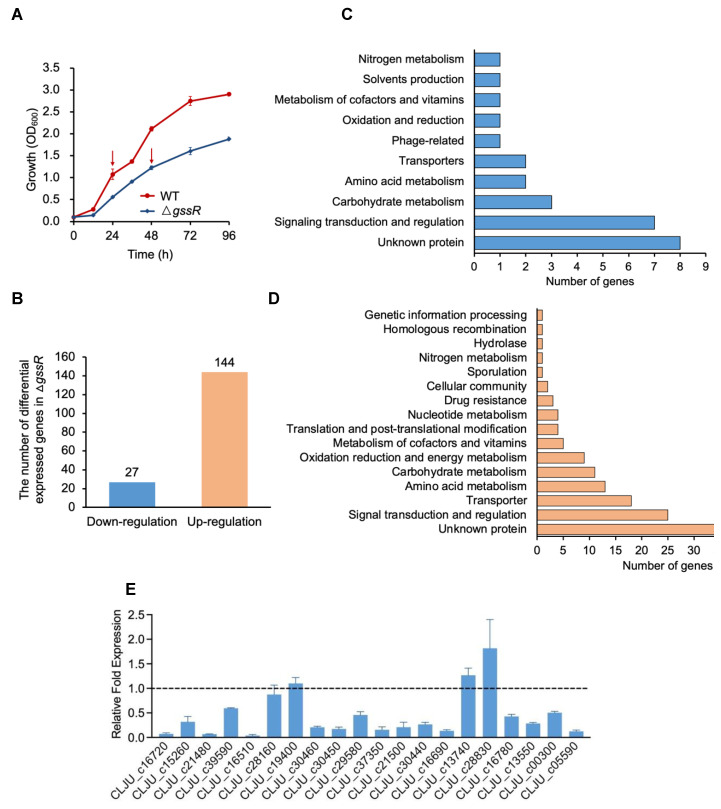

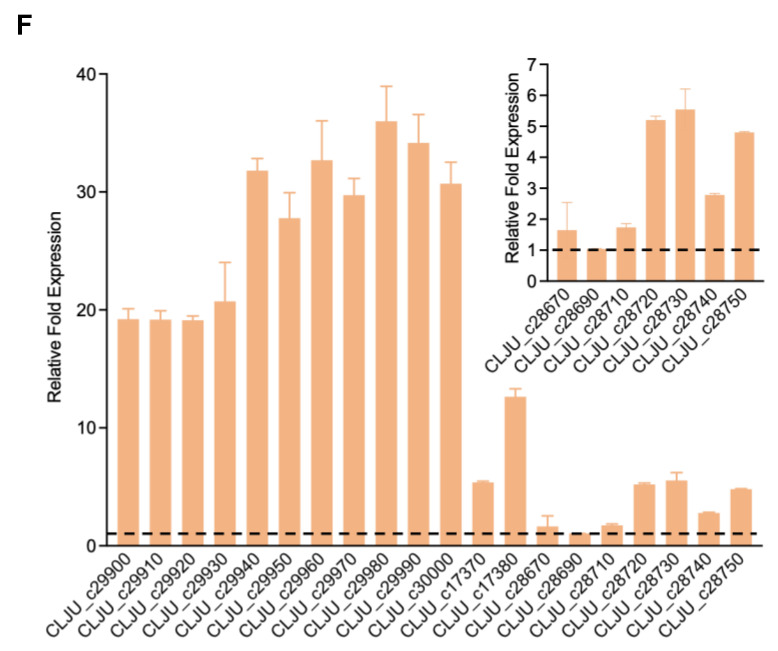
Comparative transcriptomic analysis revealed genes affected by GssR in *C. ljungdahlii*. (**A**) Changed cell growth derived from the deletion of *gssR*. The arrows reflect the sampling time points (24 h and 48 h) for RNA-seq. (**B**) The number of genes with significantly changed transcription after the deletion of *gssR*. (**C**) Functional categories of the 27 genes that were significantly down-regulated (fold change ≤ 5) after *gssR* deletion. (**D**) Functional categories of the 144 genes that were significantly up-regulated (fold change ≥ 10) after *gssR* deletion. (**E**,**F**) Validation of the RNA-seq data via real-time qRT-PCR analysis. A total of 20 up-regulated (**E**) and 20 down-regulated (**F**) genes in the *gssR*-deleted strain were selected for RT-qPCR analysis. The data are represented as mean ± standard deviation (SD) of two independent biological replicates. Error bars show SDs.

To explore the regulatory network of GssR, comparative transcriptomic analysis was conducted for mining of potential genes subjected to the regulation of GssR. Thus, both the *gssR*-deleted strain (Δ*gssR*) and the wild-type (WT) strain were grown on syngas, and the samples for RNA-seq analysis were harvested when the cell density reached OD_600_ of ~1.0 (Figure 2A).

The results of RNA-seq analysis show that the deletion of *gssR* led to significant changes in the whole gene expression profile. A large number of genes exhibited significant changes in transcription, in which the number of greatly down-regulated (5-fold) and up-regulated (10-fold) genes reached 27 and 144, respectively (Figure 2B). Among the 27 down-regulated genes, 7 were associated with signal transduction and regulation, including the Fur and BlaI-family transcriptional factors as well as the carbon starvation proteins (Figure 2C and Supplementary Table S4). Additionally, multiple genes were related to fundamental metabolism, such as carbon metabolism, amino acid metabolism, nitrogen metabolism, and energy metabolism (Figure 2C and Supplementary Table S4). Similar findings also existed for the 144 up-regulated genes, in which a lot of genes were associated with signal transduction and regulation, carbon metabolism, amino acid metabolism, energy metabolism, and substance transport (Figure 2D and Supplementary Table S5).

To validate the reliability of the RNA-seq data, 20 up-regulated and 20 down-regulated genes in the *gssR*-deleted strain were selected for RT-qPCR analysis. The results show that 35 genes exhibited similar transcriptional changes to those from RNA-seq analysis (Figure 2E,F), confirming the reliability of the RNA-seq data.

### 3.3. Identification of the Direct Targets of GssR

**Figure 3:**
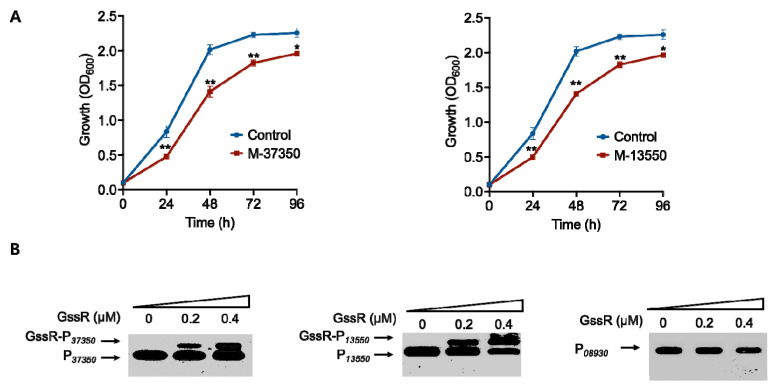
Identification of the CLJU_c37350 gene and the CLJU_c13550 gene that were directly regulated by GssR. (**A**) Influence of the repression of CLJU_c37350 (M−37350) or CLJU_c13550 (M-13550) on the growth of *C. ljungdahlii* in gas fermentation. The data are represented as mean ± standard deviation (SD) (*n* = 3). Error bars show SDs. Statistical analysis was performed using a two-tailed Student’s *t*-test. *, *p* < 0.05; **, *p* < 0.01; versus the wild-type strain containing a blank plasmid (control). (**B**) Binding of GssR to the promoter regions of the CLJU_c37350 and CLJU_c13550 genes. The probe containing the promoter region of the CLJU_c08930 gene (P*_08930_*) was used as a negative control.

The analysis of the RNA-seq data indicated that GssR is a pleiotrophic regulator in *C. ljungdahlii*. Thus, we sought to identify crucial genes that are directly regulated by GssR. Here, 28 candidate genes that are predicted to be associated with carbon assimilation and autotrophic growth of *C. ljungdahlii* were chosen for investigation (Table 1). Among them, five down-regulated genes after the deletion of *gssR* were firstly picked out for testing (Table 1). For a rapid screening, these genes were separately repressed via CRISPRi using a CRISPR-ddCas12a construct established in our laboratory, and the resulting strains were used to examine their growth in gas fermentation. As shown in Figure 3A, the M-37350 and M-13550 strains, with the repression of CLJU_c37350 or CLJU_c13550, respectively, exhibited much slower growth rate compared to the wild-type strain, while no significant change was observed for the other three strains (Supplementary Figure S1).

We further examined whether GssR can directly control the expression of CLJU_c37350 or CLJU_c13550. The results of EMSA showed that GssR exhibited varying degrees of binding to the upstream non-coding sequences of CLJU_c37350 and CLJU_c13550 (Figure 3B), suggesting that these two genes are the targets subjected to GssR’s direct regulation. According to the genome annotation, the CLJU_c37350 gene encodes CstA, a carbon starvation protein (membrane protein) that is involved in polypeptide absorption and pyruvate transport during carbon starvation [34,35]; the CLJU_c13550 gene encodes a BlaI family transcription factor, which is involved in the regulation of antibiotic tolerance and sensitivity to antimicrobial peptides [36].

**Figure 4:**
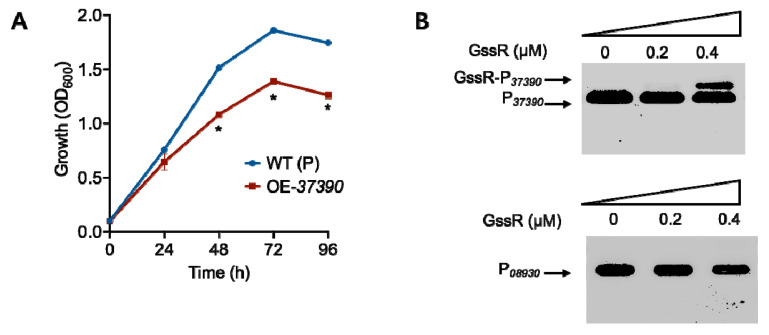
Identification of the CLJU_c37390 gene that was directly regulated by GssR. (**A**) Influence of the overexpression of CLJU_c37390 (OE-37390) on the growth of *C. ljungdahlii* in gas fermentation. The data are represented as mean ± standard deviation (SD) (*n* = 3). Error bars show SDs. Statistical analysis was performed using a two-tailed Student’s *t*-test. *, *p* < 0.05; versus WT (P) (the wild-type strain containing a blank plasmid). (**B**) Binding of GssR to the promoter regions of the CLJU_c37390 gene. The probe containing the promoter region of the CLJU_c08930 gene (P*_08930_*) was used as a negative control.

Among the remaining 23 genes that showed up-regulated transcriptional levels after the deletion of *gssR* (Table 1), 10 genes are unlikely to be related to each other, but the other 13 genes (Table 1, No. 16–28) belong to two gene clusters that are involved in the metabolism of glutamate and purines. Purines and their derivatives, as the major components of nucleotides, are necessary for energy conservation and signal transduction (GTP) in cells [37]. Here, we firstly overexpressed the former 10 genes (Table 1, No. 6–15) to examine phenotypic outcomes. Among these candidates, only the overexpression of CLJU_c37390 significantly affected the autotrophic growth of *C. ljungdahlii* (Figure 4A), while the others had no influence on cell growth (Supplementary Figure S2). According to the genome annotation, the CLJU_c37390 gene encodes a pyruvate carboxylase, which is known to be responsible for the conversion of pyruvate to oxaloacetate at the expense of ATP [38].

Based on this finding, we further examined the interaction between GssR and the CLJU_c37390 gene using EMSA. Specifically, a 305 bp DNA fragment covering the promoter region of the CLJU_c37390 gene was used as the DNA probe. As shown in Figure 4B, GssR yielded a clear mobility shift at a lower protein concentration (0.4 µM), whereas no evident mobility shift was observed for the DNA probe P*_08930_* (negative control) at the same protein concentration. This indicated that GssR could directly regulate the expression of CLJU_c37390.

**Figure 5:**
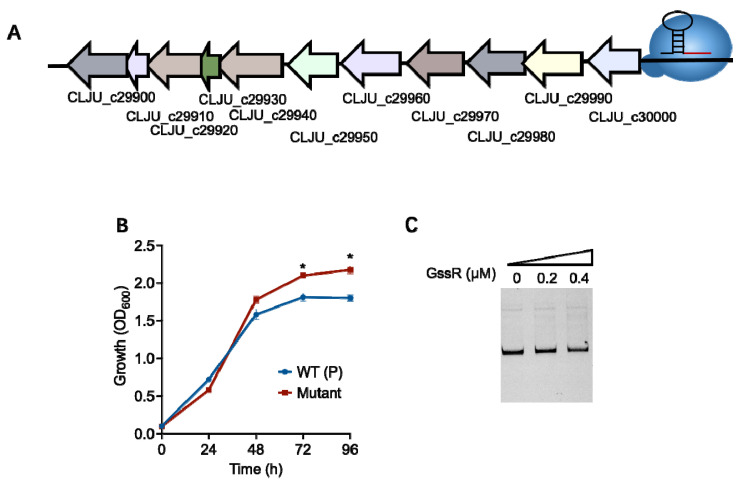
The regulatory effect of GssR on the gene cluster (CLJU_c29900–30000) involved in purine metabolism. (**A**) Schematic diagram of the repression of the gene cluster (CLJU_c29900–30000) by CRISPRi in *C. ljungdahlii*. The data are represented as mean ± standard deviation (SD) (*n* = 3). Error bars show SDs. Statistical analysis was performed using a two-tailed Student’s *t*-test. *, *p* < 0.05; versus WT (P) (the wild-type strain containing a blank plasmid). (**B**) The influence of the repression of the gene cluster (CLJU_c29900–30000) on the growth of *C. ljungdahlii*. (**C**) Electrophoretic mobility shift assay (EMSA) analysis using the purified GssR protein and the probe containing the promoter region of the purine metabolism gene cluster. Binding of GssR to the probe was not observed.

Regarding the abovementioned gene clusters which are involved in the metabolism of glutamate and purines, we chose to repress their expression by using a CRISPR-ddCas12a construct that targeted their upstream promoter regions (Figure 5A; Supplementary Figure S3A) because the deletion or overexpression of the whole cluster in *C. ljungdahlii* is difficult. As shown in Supplementary Figure S3B, the repression of the gene cluster (CLJU_c17370–17380) for glutamate metabolism did not cause significant change in cell growth; in contrast, the repression of the gene cluster (CLJU_c29900–30000) for purine metabolism could enhance the growth of *C. ljungdahlii* in gas fermentation, achieving an increase of 21.7% in the final biomass compared to that of the control strain (Figure 5B). However, the following EMSA showed no binding of GssR to the promoter region of the latter cluster (Figure 5C), suggesting an indirect regulation of GssR on this gene cluster.

### 3.4. Direct Binding of BirA on gssR

**Figure 6:**
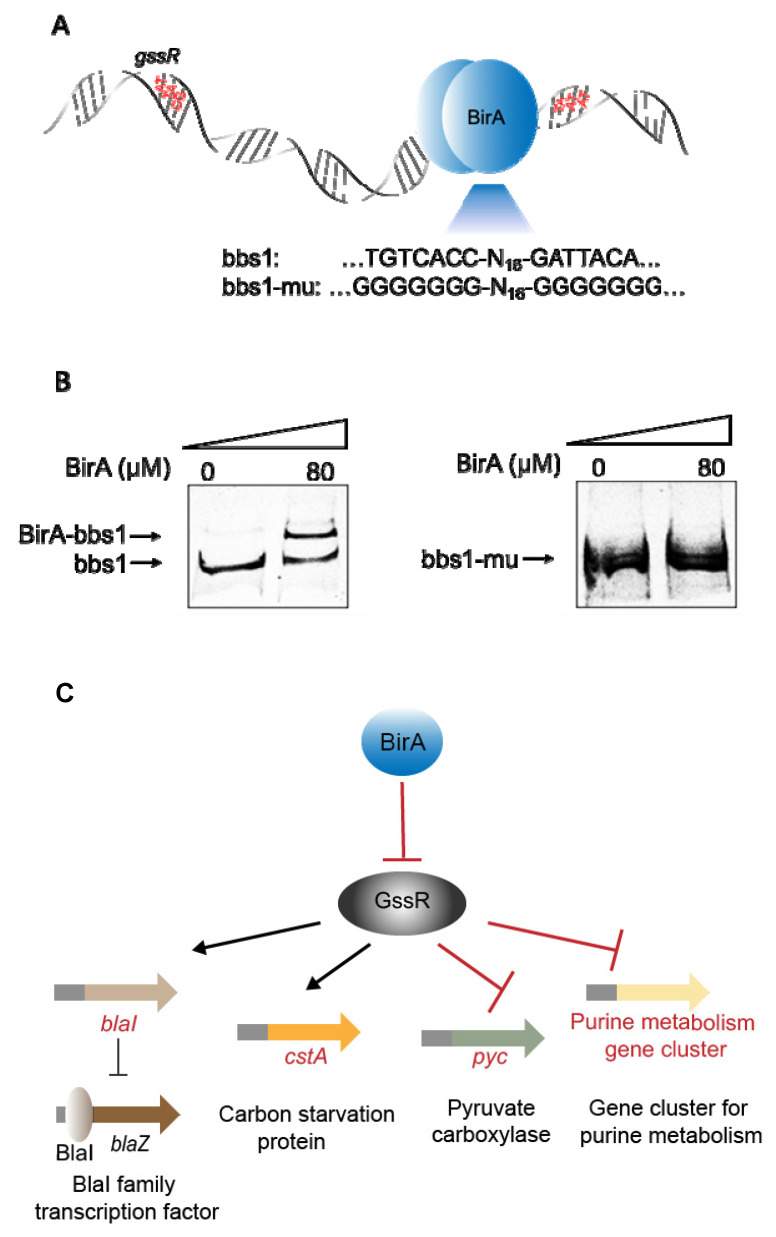
The binding of BirA to the *gssR* gene. (**A**) The putative BirA-binding site (bbs1) within the coding region of *gssR*. The bbs1 and its mutant (bbs1-mu, with the mutation of the 7 nt reverse repeats at two ends of bbs1 to “GGGGGGG”) were used for the following EMSA analysis. (**B**) EMSA analysis confirming the binding of the purified BirA protein to the coding region of *gssR*. BirA could bind to the DNA probe containing the bbs1 sequence (**left** panel), but the binding was disrupted when bbs1 was replaced with bbs1-mu (**right** panel). (**C**) Schematic diagram of a possible multi-level regulatory network that employs both BirA and GssR in *C. ljungdahlii*.

We previously identified a bifunctional protein, BirA, that is involved in the regulation of carbon assimilation in *C. ljungdahlii* [14]. This protein functions as both a biotin ligase and a transcriptional factor [39]. Here, based on the data from the ChIP-seq analysis in our lab [14], we detected an enrichment of BirA in the coding region of the *gssR* gene; more importantly, according to the reported BirA-binding motif [40], a highly similar sequence bbs1 (TGTCACC-N_16_-GATTACA) was found within the coding region of *gssR*. These findings strongly indicated that BirA could directly regulate the expression of *gssR*. To investigate this possibility, we performed an EMSA analysis by using the purified BirA protein and a DNA probe that contained the bbs1 sequence. As expected, BirA could bind to the DNA probe, whereas the mutation at the two ends of bbs1 disrupted the binding (Figure 6A,B). Given that the BirA-binding site was within the coding region of *gssR*, BirA appears to play a negative regulating action on *gssR*.

These results reveal a regulatory mechanism mediated by both the BirA and GssR in *C. ljungdahlii*, in which BirA may directly control the expression of GssR and GssR further regulates the downstream genes and pathways (Figure 6C), consequently affecting the strain performance in gas fermentation.

### 3.5. GssR Evolutionary Tree and Universality Analysis

**Figure 7:**
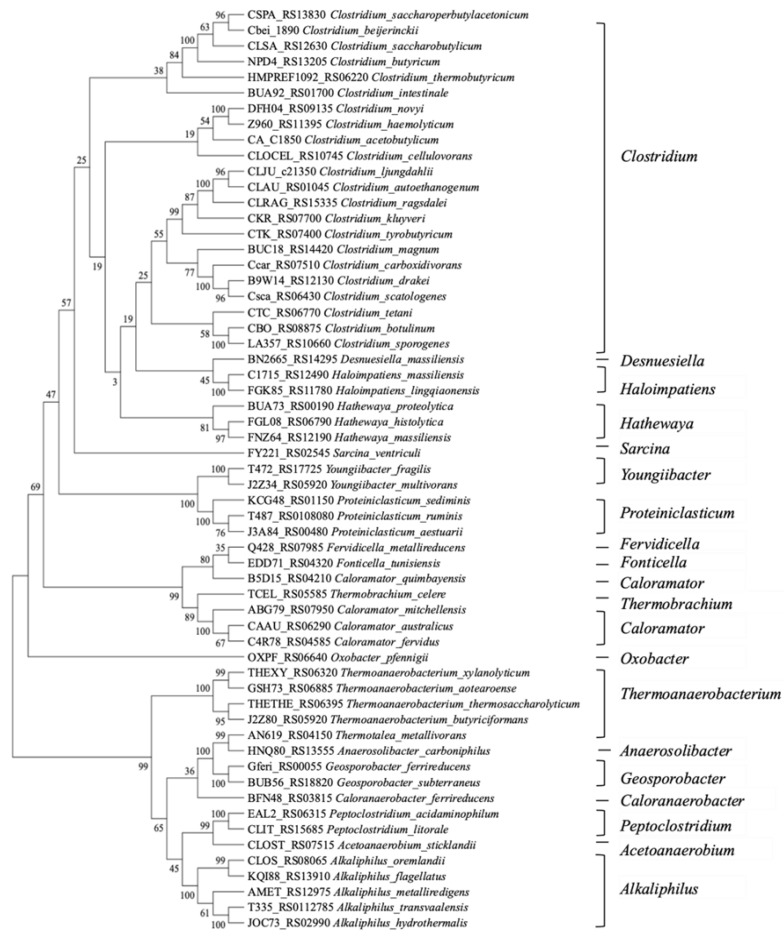
Maximum likelihood phylogenetic tree of GssR homologues in bacteria.

The *C. ljungdahlii* GssR belongs to the RpiR family; however, it exhibits limited similarity with the previously reported transcription factors of this family. To explore the evolutionary relationship of GssR-like proteins and the distribution of GssRs in different bacterial strains, we performed amino acid sequence alignments and analysis. Interestingly, we discovered GssR homologues in numerous bacterial species. Thus, 58 highly conserved homologues (>63% sequence identity) were selected to construct a phylogenetic tree using maximum likelihood phylogenetic analysis (Figure 7). The results reveal that GssR and its homologues are predominantly found in anaerobic bacteria, including *Clostridium*, *Desnuesiella*, *Haloimpatiens*, *Hathewaya*, *Sarcina*, *Youngiibacter*, *Proteiniclasticum*, *Fervidicella*, *Caloramator*, *Thermobrachium*, *Caloramator*, *Oxobacter*, *Thermoanaerobacterium*, *Geosporobacter*, *Peptoclostridium*, and *Alkaliphilus*, and especially widely distributed in *Clostridium,* with high amino acid sequence similarity. The sequence similarity of GssR proteins is more consistent with the phylogeny of these species.

**Figure 8:**
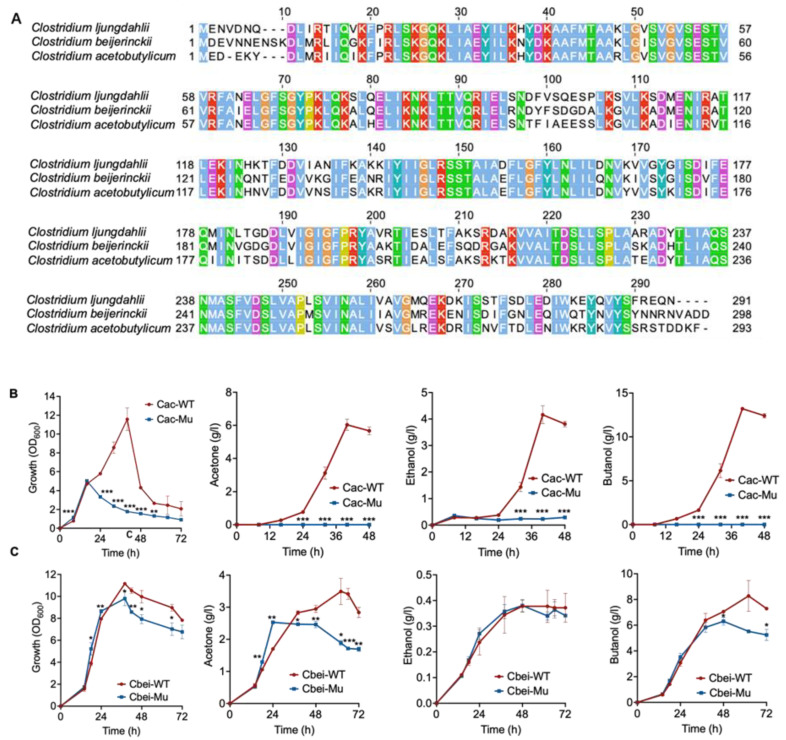
Sequence alignment of GssR proteins of three *Clostridium* species and phenotypic outcomes of the *gssR* deletion in *C. acetobutylicum* and *C. beijerinckii*. (**A**) Sequence alignment of the GssR proteins. The genes coding for the *C. acetobutylicum* and *C. beijerinckii* GssRs is CAC1850 and Cbei1890, respectively. (**B**) Influence of the inactivation of the CAC1850 gene on the growth and solvent production of *C. acetobutylicum* ATCC 824. Cac-WT: the wild-type strain. Cac-Mu: the mutated strain. (**C**) Influence of the inactivation of the Cbei1890 gene on the growth and solvent production of *C. beijerinckii* NCIMB 8052. Cbei-WT: the wild-type strain. Cbei-Mu: the mutated strain. The data are represented as mean ± standard deviation (SD) (*n* = 3). Error bars show SDs. Statistical analysis was performed using a two-tailed Student’s *t*-test. *, *p* < 0.05; **, *p* < 0.01; ***, *p* < 0.001; versus the wild-type strain.

Specific to the two representative solventogenic *Clostridium* species, *C. acetobutylicum* and *C. beijerinckii*, their GssR homologs showed high amino acid sequence similarity (80.07% and 75%, respectively) to that of *C. ljungdahlii* (Figure 8A). To examine the importance of these two proteins in their hosts, we inactivated their corresponding genes, i.e., CAC1850 and Cbei1890, by using the Targetron method [41], yielding the mutant strains Cac-Mu and Cbei-Mu, respectively, to examine their performance in fermentation.

The results show that the growth of the Cac-Mu was substantially impaired compared to the wild-type strain (Figure 8B). Consequently, the production of acetone and ethanol also decreased, and moreover, almost no butanol production was detected for the mutant strain (Figure 8B). Simultaneously, the other mutant strain, Cbei-Mu, also exhibited impaired growth and reduced production of acetone and butanol (Figure 8C); in contrast, its ethanol production had no significant changes compared to the wild-type strain (Figure 8C). Taken together, these results demonstrate that GssR also plays crucial regulatory roles in saccharolytic *C. acetobutylicum* and *C. beijerinckii*, indicating the universality of its function in *Clostridium* species.

## 4. Discussion

The RpiR family transcription factors have been known to regulate the consumption of multiple carbon sources. For example, RpiR was found to regulate the expression of ribose phosphate isomerase in *Escherichia coli*, thereby affecting central carbon metabolism [42]; in *Bacillus subtilis*, GlvR acted as a positive regulator of maltose metabolism [43]; the *Neisseria meningitidis* HexR is involved in repressing glucose metabolism [44]. Despite these previous findings, the roles of RpiR family transcription factors in autotrophic acetogens remain unexplored. In this study, we demonstrated that GssR plays a pleiotropic regulator role in *C. ljungdahlii* and exerts a positive regulation on cell growth and product synthesis. Enhancement of the *gssR* expression could promote the strain growth and the product synthesis in gas fermentation. Considering the wide distribution of GssR-like regulators in anaerobic bacteria, especially *Clostridium* species, it may serve as a potential target for further strain modification and improvement.

The comparative transcriptomic analysis indicated that GssR could modulate the expression of a large number of genes in *C. ljungdahlii*. Many of them are associated with signal transduction, carbon metabolism, substance transport, and amino acid metabolism, which play critical roles in the maintenance of physiological functions in bacteria [45,46]. For gas-fermenting *Clostridium* species, the Rnf complex has been known to be responsible for proton transport and ATP synthesis in *C. ljungdahlii*, and the disruption of the *rnf* operon significantly impaired cell growth [47]. Here, GssR was found to repress the expression of a gene cluster responsible for purine metabolism in *C. ljungdahlii*. Purines and their derivatives are known to exert important physiological functions in microorganisms because they are not only the major components of nucleotides but also the crucial carriers in energy conservation and signal transduction [37]. Thus, we speculated that GssR can inhibit purine metabolism, consequently increasing the supply of purine in *C. ljungdahlii*. This would enhance energy conservation and nucleotide synthesis in cells, thereby promoting stain growth.

It is noteworthy that, among the genes subjected to GssR’s regulation, some are located in the WLP, including *fdh* (CLJU_c06990 and CLJU_c20040), *metF* (CLJU_c37610 and CLJU_c37630), and *codH* (CLJU_c17910), which were up-regulated by 3.15, 2.3, 3.13, 4.07, and 2.43-fold, respectively, after the deletion of *gssR* (Table 2). Therefore, it seems that GssR may negatively regulate the carbon fixation pathway in *C. ljungdahlii*. Additionally, we found that the CLJU_c37390 gene, encoding a pyruvate carboxylase, was directly regulated by GssR (Figure 4). Pyruvate carboxylase is known to catalyze the formation of oxaloacetate from pyruvate and CO_2_, thereby serving as a key enzyme responsible for CO_2_ fixation in microbes [48]. Thus, GssR may also affect CO_2_ fixation in *C. ljungdahlii* through the regulation of pyruvate carboxylase. However, based on the genomic information, *C. ljungdahlii* has an incomplete TCA cycle in which several key enzymes, such as succinyl-CoA synthetase and malate dehydrogenase, are missing [38]. Hence, the conversion of oxaloacetate that comes from pyruvate and CO_2_ may depend on other pathways in *C. ljungdahlii*.

An interesting finding is that GssR was directly controlled by another transcription factor BirA in *C. ljungdahlii*, which probably forms a cascade regulatory pathway. BirA is a well-known regulator in bacteria and is involved in the regulation of multiple biological processes [40]. Our previous study also revealed the significance of BirA in the regulation of carbon fixation in *C. ljungdahlii* [14]. Here, we discovered that BirA can bind to the coding region of *gssR* (Figure 6B), probably exerting an inhibitory effect on GssR’s function. Such a regulatory pathway employing two regulators, to our knowledge, has not been reported in autotrophic acetogens. However, the biological significance of this multilevel regulatory pathway in *C. ljungdahlii* is still unclear and merits further exploration.

In summary, this work showcases the importance of GssR in gas-fermenting *C. ljungdahlii*. Based on this finding, we further elucidated the pleiotropic regulatory functions of GssR in this bacterium. Furthermore, a potential regulatory pathway mediated by both GssR and its upstream regulator BirA was generated. These findings provide new insights into the significance and application value of transcription factors in gas-fermenting *Clostridium* species.

## Figures and Tables

**Table 1:** The 28 candidate genes chosen for mining of the regulatory targets of GssR.

Number	Locus Tag	Function	^a^ Log_2_ Fold Changes	Pathways
1	CLJU_c15260	predicted NADPH-flavin oxidoreductase	−2.41	Oxidation and reduction
2	CLJU_c21480	aminomethyltransferase	−2.72	Amino acid metabolism
3	CLJU_c21500	glutamate formiminotransferase	−3.67	Amino acid metabolism
4	CLJU_c37350	carbon starvation protein A	−3.33	Signaling transduction and regulation
5	CLJU_c13550	BlaI family transcriptional regulator	−3.99	Signaling transduction and regulation
6	CLJU_c03860	putative D-isomer specific 2-hydroxyacid dehydrogenase family protein	3.32	Amino acid metabolism
7	CLJU_c21310	S-adenosylmethionine decarboxylase	3.48	Amino acid metabolism
8	CLJU_c24380	cysteine-S-conjugate beta-lyase	3.83	Amino acid metabolism
9	CLJU_c20510	5-oxoprolinase (ATP-hydrolysing) subunit A	4.57	Amino acid metabolism
10	CLJU_c20480	urea carboxylase	4.98	Hydrolase
11	CLJU_c37390	pyruvate carboxylase	3.69	Carbohydrate metabolism
12	CLJU_c06630	isocitrate dehydrogenase (NAD^+^)	4.36	Carbohydrate metabolism
13	CLJU_c25360	malate dehydrogenase (oxaloacetate-decarboxylating)	4.10	Carbohydrate metabolism
14	CLJU_c15270	(S)-ureidoglycine aminohydrolase	4.16	Nucleotide metabolism
15	CLJU_c22770	biotin synthase	3.59	Metabolism of cofactors and vitamins
16	CLJU_c17370	putative D-isomer specific 2-hydroxyacid dehydrogenase family protein	3.67	Amino acid metabolism
17	CLJU_c17380	S-adenosylmethionine decarboxylase	4.56	Amino acid metabolism
18	CLJU_c29900	adenine deaminase	3.82	Nucleotide metabolism
19	CLJU_c29910	putative purine deaminase, zinc-binding domain	6.33	Nucleotide metabolism
20	CLJU_c29920	putative xanthine dehydrogenase subunit, FAD-binding domain	4.34	Oxidation and reduction
21	CLJU_c29930	putative oxidoreductase, iron-sulfur binding subunit	5.30	Oxidation and reduction
22	CLJU_c29940	xanthine dehydrogenase related protein, molybdopterin bindin	5.36	Oxidation and reduction
23	CLJU_c29950	putative deacetylase	5.39	Post-translational modification
24	CLJU_c29960	N-acyl-D-amino-acid deacylase	4.61	Post-translational modification
25	CLJU_c29970	predicted ABC-type transporter, permease component	3.70	Transporter
26	CLJU_c29980	predicted ABC-type transporter, permease component	3.96	Transporter
27	CLJU_c29990	predicted ABC-type transporter, permease component	3.97	Transporter
28	CLJU_c30000	putative basic membrane protein	3.25	Transporter

^a^ Mean data of two biological replicates.

**Table 2:** Transcriptional changes of five genes located in WLP after the deletion of *gssR*.

Locus Tag	Function	^a^ Log_2_ Fold Changes
CLJU_c06990	formate dehydrogenase, alpha subunit	1.66
CLJU_c20040	formate dehydrogenase, alpha subunit	1.20
CLJU_c37610	methylenetetrahydrofolate reductasen	1.65
CLJU_c37630	methylenetetrahydrofolate dehydrogenase	2.02
CLJU_c17910	carbon monoxide dehydrogenase	1.28

^a^ the mean data of two biological replicates.

## Data Availability

The data presented in this study are available on request from the corresponding authors.

## Supplementary Materials

The following supporting information can be downloaded at https://www.mdpi.com/article/10.3390/microorganisms11081968/s1. Figure S1: Influence of the repression of CLJU_c15260 (M-315260), CLJU_c21480 (M-21480), or CLJU_c21500 (M-21500) on the growth of *C. ljungdahlii* in gas fermentation; Figure S2: Influence of the overexpression of nine genes that showed up-regulated transcriptional levels after the deletion of *gssR* on the growth of *C. ljungdahlii* in gas fermentation; Figure S3: Influence of the repression of the gene cluster (CLJU_c17370–17380) for glutamate metabolism on the growth of *C. ljungdahlii* in gas fermentation; Table S1: Strains used in this study; Table S2: Plasmids used in this study; Table S3: Primers used in this study. Table S4: 27 genes with significantly altered transcriptional level (change fold ≤ 5.0) after the deletion of gssR (ΔgssR/Wild Type). Control: Wild Type. Experiment: ΔgssR. The data were the mean of two biological replicates. Table S5: 144 genes with significantly altered transcriptional level (change fold ≥ 10.0) after the deletion of gssR (ΔgssR/Wild Type). Control: Wild Type. Experiment: ΔgssR. The data were the mean of two biological replicates.
