# Supplementary material for: Pleiotropic Regulator GssR Positively Regulates Autotrophic Growth of Gas-Fermenting Clostridium ljungdahlii
Source: Microorganisms. 2023 Jul 31;11(8):1968. doi: 10.3390/microorganisms11081968 (PMC10458427; doi:10.3390/microorganisms11081968)
Supplement: Supplementary file 1 [file microorganisms-11-01968-s001.zip › GssR-supplementary Data.pdf]

Microorganisms

Supplementary Data

Pleiotropic Regulator GssR Positively Regulates Autotrophic Growth of Gas-Fermenting *Clostridium ljungdahlii*

Huan Zhang [1,2,†], Can Zhang [1,2,†], Xiaoqun Nie [1,2,], Yuwei Wu [1,2], Chen Yang [1], Weihong Jiang [1,*] and Yang Gu [1,*]

[1] CAS-Key Laboratory of Synthetic Biology, CAS Center for Excellence in Molecular Plant Sciences,
Shanghai Institute of Plant Physiology and Ecology, Chinese Academy of Sciences, 300 Fenglin Road, Shanghai 200032, China
[2] University of Chinese Academy of Sciences, Beijing 100049, China
* Correspondence: wjiang@cemps.ac.cn (W.J.); ygu@cemps.ac.cn (Y.G.); Tel: +86-21-54924172 (W.J.);
+86-21-54924284 (Y.G.); Fax: +86-21-54924015 (W.J. & Y.G.)
† These authors contributed equally to this work.

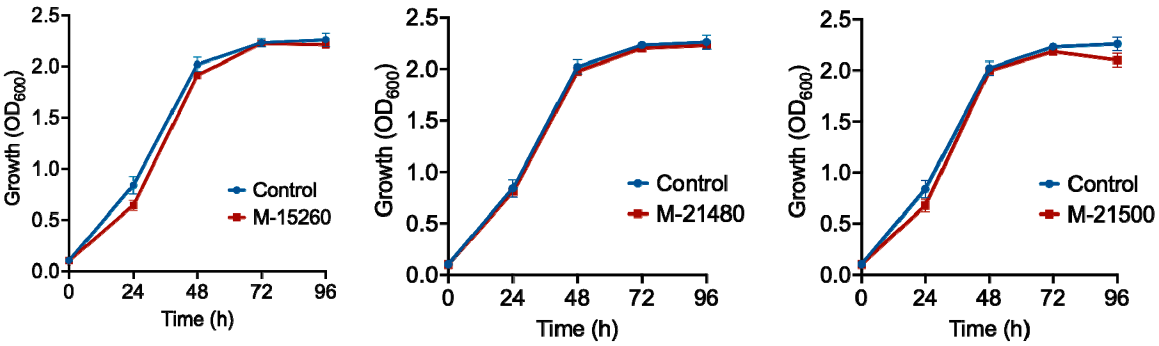

Supplementary Figure S1. Influence of the repression of CLJU_c15260 (M-315260), CLJU_c21480 (M-21480), or CLJU_c21500 (M-21500) on the growth of *C. ljungdahlii* in gas fermentation. The data are represented as mean ± standard deviation

(SD) (n = 3). Error bars show SDs. Statistical analysis was performed by a two-tailed Student's *t*-test.

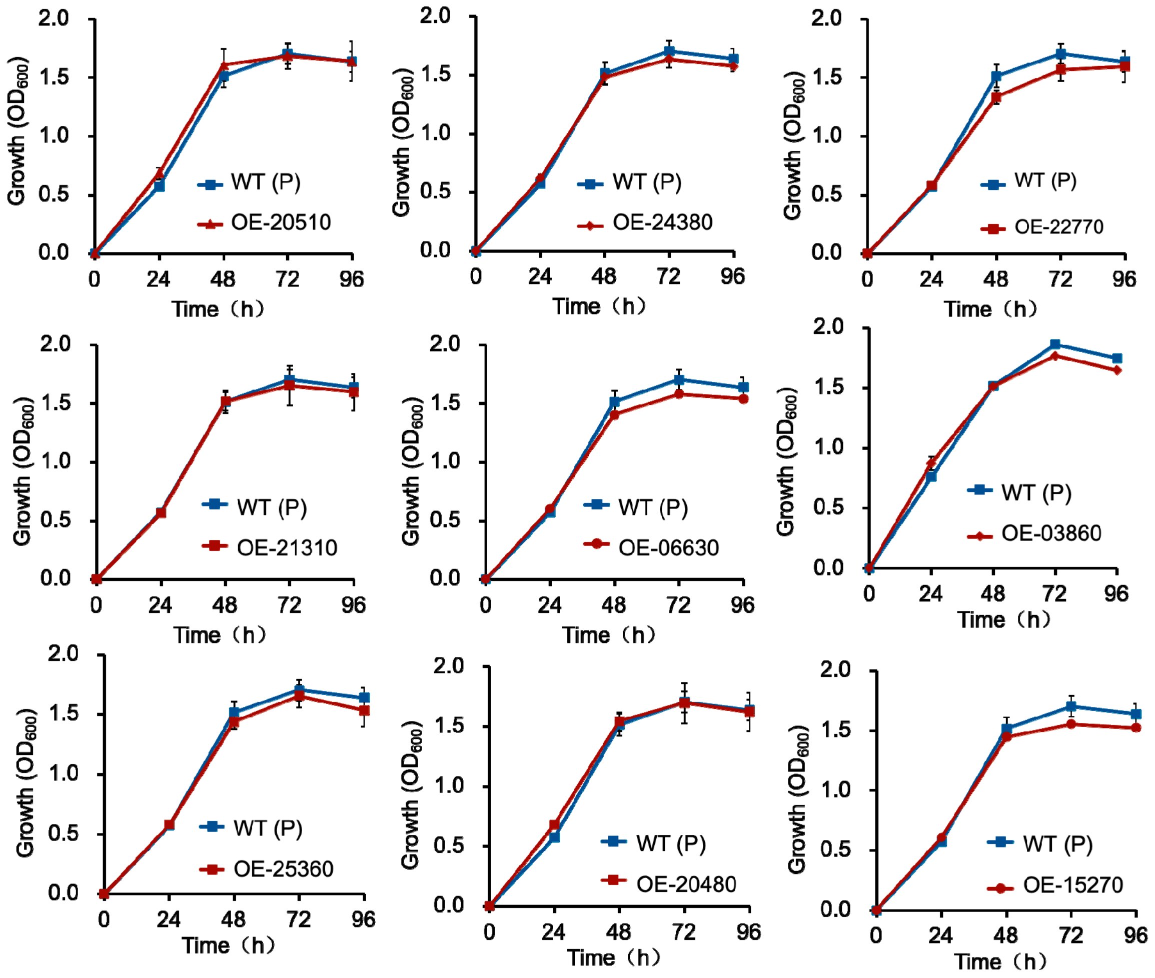

Supplementary Figure S2. Influence of the overexpression of nine genes that showed up-regulated transcriptional levels after the deletion of *gssR* on the growth of *C. ljungdahlii* in gas fermentation. The data are represented as mean ± standard deviation (SD) (n = 3). Error bars show SDs. Statistical analysis was performed by a two-tailed Student's t-test.

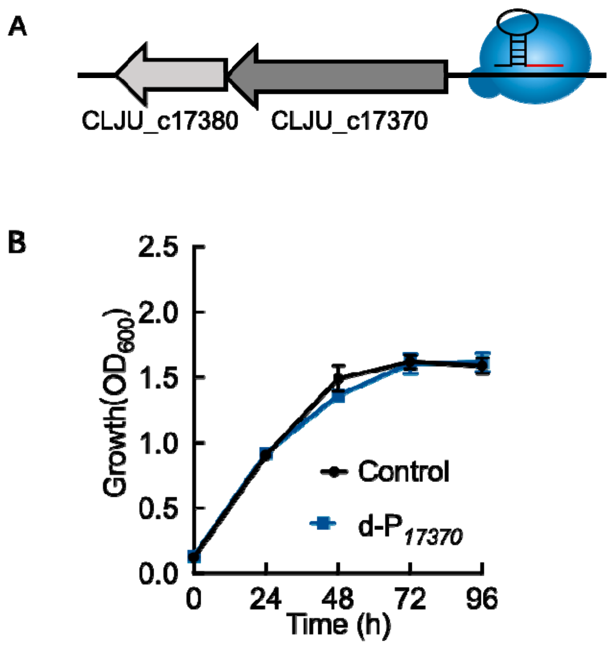

Supplementary Figure S3. Influence of the repression of the gene cluster (CLJU_c17370–17380) for glutamate metabolism on the growth of *C. ljungdahlii* in gas fermentation.

Supplementary Table S1. Strains used in this study

Strains	Description of genotypes	Source
**Strains**		
E. coli		
BL21 (DE3)	Strain used for protein overexpression	Novagen
DH5α	General cloning host strain	Invitrogen
C. ljungdahlii		
WT (P)	Wild-type *C. ljungdahlii*, carrying the pMTL-83151 plasmid	This study
△*gssR* (P)	Wild-type *C. ljungdahlii* with the deletion of *gssR* (CLJU_c21350), carrying the pMTL-83151 plasmid	This study
△*gssR*-*gssR*	Wild-type *C. ljungdahlii* with the deletion of *gssR*, carrying the pMTL-P_{gssR}-*gssR* plasmid	This study
P_{gssR}-*gssR*	Wild-type *C. ljungdahlii*, carrying the pMTL-P_{gssR}-*gssR* plasmid	This study
WT	Wild-type *C. ljungdahlii*, DSM 13528	DSMZ
△*gssR*	Wild-type *C. ljungdahlii* with the deletion of *gssR*	This study
Control	Wild-type *C. ljungdahlii*, carrying the pZG-ddFncas12a plasmid	This study
d-37350	Wild-type *C. ljungdahlii*, carrying the pZG-ddFncas12a-37350 plasmid	This study
d-13550	Wild-type *C. ljungdahlii*, carrying the pZG-ddFncas12a-13550 plasmid	This study
d-15260	Wild-type *C. ljungdahlii*, carrying the pZG-ddFncas12a-15260 plasmid	This study
d-21480	Wild-type *C. ljungdahlii*, carrying the pZG-ddFncas12a-21480 plasmid	This study
d-21500	Wild-type *C. ljungdahlii*, carrying the pZG-ddFncas12a-21500 plasmid	This study
d-P_{30000}	Wild-type *C. ljungdahlii*, carrying the pZG-ddFncas12a- P_{30000} plasmid	This study
d-P_{17370}	Wild-type *C. ljungdahlii*, carrying the pZG-ddFncas12a- P_{17370} plasmid	This study
WT (P)	Wild-type *C. ljungdahlii*, carrying the pMTL-83151 plasmid	This study
P_{37390}-37390	Wild-type *C. ljungdahlii*, carrying the pMTL83151- P_{37390}-37390 plasmid	This study
P_{20510}-20510	Wild-type *C. ljungdahlii*, carrying the pMTL83151- P_{20510}-20510 plasmid	This study

P_{24380}-24380	Wild-type *C. ljungdahlii*, carrying the pMTL83151- P_{24380}-24380 plasmid	This study
P_{22770}-22770	Wild-type *C. ljungdahlii*, carrying the pMTL83151- P_{22770}-22770 plasmid	This study
P_{21310}-21310	Wild-type *C. ljungdahlii*, carrying the pMTL83151- P_{21310}-21310 plasmid	This study
P_{06630}-06630	Wild-type *C. ljungdahlii*, carrying the pMTL83151- P_{06630}-06630 plasmid	This study
P_{03860}-03860	Wild-type *C. ljungdahlii*, carrying the pMTL83151- P_{03860}-03860 plasmid	This study
P_{25360}-25360	Wild-type *C. ljungdahlii*, carrying the pMTL83151- P_{25360}-25360 plasmid	This study
P_{20480}-20480	Wild-type *C. ljungdahlii*, carrying the pMTL83151- P_{20480}-20480 plasmid	This study
P_{15270}-15270	Wild-type *C. ljungdahlii*, carrying the pMTL83151- P_{15270}-15270 plasmid	This study
C. acetobutylicum		
Cac	*C. acetobutylicum* ATCC 824	ATCC
Cac-Mu	The *C. acetobutylicum* mutant with *CAC1850*::intron	This study
C. beijerinckii		
Cbei	*C. beijerinckii* NCIMB 8052	NCIMB
Cbei-Mu	The *C. beijerinckii* mutant with *Cbei1890*::intron	This study

Supplementary Table S2. Plasmids used in this study

plasmids	Description of genotypes	Source
pMTL83151	*ColE1, catP, pCB102 ori*	Provided by Prof. Nigel P. Minton [21]
pMTLcas-*pta*	*pCB102 ori, catP, ColE1, tra,* P_{thl}-*cas9,* P_{araE}-*sgRNA,* homologous arms for *pta*	This lab [20]
pMTLcas-*gssR*	*ColE1, catP, pCB102 ori,* P_{thl}-*cas9,* P_{1339}-*sgRNA,* homologous arms for *gssR*	This study
pMTL83151- P_{gssR}-*gssR*	*gssR* overexpression, derived from pMTL83151	This study
pMTL83151- P_{37390}-37390	CLJU_c37390 overexpression, derived from pMTL83151	This study
pMTL83151- P_{20510}-20510	CLJU_c20510 overexpression, derived from pMTL83151	This study
pMTL83151- P_{24380}-24380	CLJU_c24380 overexpression, derived from pMTL83151	This study
pMTL83151- P_{22770}-22770	CLJU_c22770 overexpression, derived from pMTL83151	This study
pMTL83151- P_{21310}-21310	CLJU_c21310 overexpression, derived from pMTL83151	This study
pMTL83151- P_{06630}-06630	CLJU_c06630 overexpression, derived from pMTL83151	This study
pMTL83151- P_{03860}-03860	CLJU_c03860 overexpression, derived from pMTL83151	This study
pMTL83151- P_{25360}-25360	CLJU_c25360 overexpression, derived from pMTL83151	This study
pMTL83151- P_{20480}-20480	CLJU_c20480 overexpression, derived from pMTL83151	This study
pMTL83151- P_{15270}-15270	CLJU_c15270 overexpression, derived from pMTL83151	This study
pZG-ddFncas12a	*ColE1, catP, pCB102 ori,* P_{01440}-*Fncas12a,* P_{thl}	This lab [25]
pZG-ddFncas12a-37350	CLJU_c37350 repression vector derived from pZG-ddFncas12a	This study
pZG-ddFncas12a-13550	CLJU_c13550 repression vector derived from pZG-ddFncas12a	This study
pZG-ddFncas12a-15260	CLJU_c15260 repression vector derived from pZG-ddFncas12a	This study
pZG-ddFncas12a-21480	CLJU_c21480 repression vector derived from pZG-ddFncas12a	This study
pZG-ddFncas12a-21500	CLJU_c21500 repression vector derived from pZG-ddFncas12a	This study
pZG-ddFncas12a-P_{30000}	The vector for repressing purine metabolism,	This study

	derived from pZG-ddFncas12a	
pZG-ddFncas12a-P_{17370}	The vector for repressing glutamate metabolism, derived from pZG-ddFncas12a	This study
pWJ1	Em^r; Amp^r; *pCB102*; P_{ptb}; Group II intron	This lab [22]
pWJ1-CAC1850	For *cac1850* inactivation by intron insertion at 381, 382s site	This study
pWJ1-Cbei1890	For *cbei1890* inactivation by intron insertion at 357, 358s site	This study

Supplementary Table S3. Primers used in this study

Primer name	Sequence (5′–3′)	Description
gssR-gRNA-for	CTTAAGGAGGAGTTTTCGTCGACGCTAAATTAGGTGTGAGTGTGTTTTAGAGCTAGAAA	forward primer of *gssR* gRNA
gRNA-crRNA-rev	ATAAAAATAAGAAGCCTGCAAATGCAGGCTTCTTATTTTTATAAAAAAAGCACCGACTC	universal reverse primer of gRNA
gssR-UpArm-for	TGCAGGCTTCTTATTTTTATTAAGACAAATAAGTATACAG	forward primer of *gssR* up homologous arm
gssR-UpArm-rev	CCCTTGTAGAAAATTCATCTTTGCTTCATTCCTTTCTTTC	reverse primer of *gssR* up homologous arm
gssR-DownArm-for	AGATGAATTTTCTACAAGGG	forward primer of *gssR* down homologous arm
gssR-DownArm-rev	AAGCTTGCATGTCTGCAGGCCTCGAGGAAGCAAATTTACTAATGTATG	reverse primer of *nrdR* down homologous arm
P_{gssR}-for	TGACCGCGGCCGCTGTATCCATATGGGCATTTTCAAAGAAATAAC	forward primer used for *gssR* overexpression
G_{gssR} -rev	GTAAAACGACGGCCAGTGCCAAGCTTTTAATTTTGTTCTCTAAAAG	reverse primer used for *gssR* overexpression
P_{37390}-for	GCGGCCGCTGTATCCATATGATAAGTTATAGAGTTTCTAACTTAAAAAT	forward primer used for CLJU_c37390 overexpression
G_{37390}-rev	ACGACGGCCAGTGCCAAGCTTCTATTTTAATCTAATTAAAAGTTCTCCAG	reverse primer used for CLJU_c37390 overexpression
P_{20510}-for	GCGGCCGCTGTATCCATATGATGCATGATAAACAGAGTTCT	forward primer used for CLJU_c20510 overexpression
G_{20510}-rev	CCATATCAACATTAATATCTATCTTCATTTTTAGACCTCCTTAAATCGG	reverse primer used for CLJU_c20510 overexpression
P_{24380}-for	GCGGCCGCTGTATCCATATGATTTAAAGTGGGGCGTCAC	forward primer used for CLJU_c24380 overexpression
G_{24380}-rev	CCGTGTATTAATAATGATTCTATATTCATGATTATTGTCCTCCTTATAAATAATTTC	reverse primer used for CLJU_c24380 overexpression
P_{22770}-for	GCGGCCGCTGTATCCATATGTACATATGTCTCCTTTTGAGGT	forward primer used for CLJU_c22770 overexpression
P_{22770}-rev	CATCATTAAGGATTTTATCTATAGTCATTAGATCACCTCCTGTATTAGAT	reverse primer used for CLJU_c22770 overexpression
G_{22770}-for	ATCTAATACAGGAGGTGATCTAATGACTATAGATAAAATCCTTAATGATG	forward primer used for CLJU_c22770 overexpression
G_{22770}-rev	ACGACGGCCAGTGCCAAGCTTCTAATAATCTCTTGATATTTCATATTCT	reverse primer used for CLJU_c22770 overexpression

	G	
P_{21310}-for	GCGGCCGCTGTATCCATATGATTAAATATTTTTAATTTACTACCCTGAAG	forward primer used for CLJU_c21310 overexpression
G_{21310}-rev	ACGACGGCCAGTGCCAAGCTTTTATATATTTGAAGCTTTTACTTCTACAAC	reverse primer used for CLJU_c21310 overexpression
P_{06630}-for	GCGGCCGCTGTATCCATATGAAAATTATTCCTCCTTGAAAATTCT	forward primer used for CLJU_c06630 overexpression
G_{06630}-rev	AGAGTAACTTTATGCGTCATTTAATTCATCTCCTCGGATTAATAT	reverse primer used for CLJU_c06630 overexpression
P_{03860}-for	GCGGCCGCTGTATCCATATGGCTGATGAAAAGAGCATGTT	forward primer used for CLJU_c03860 overexpression
G_{03860}-rev	ACGACGGCCAGTGCCAAGCTTTTAAAGTTTGTTCACAATAAATTTAGG	reverse primer used for CLJU_c03860 overexpression
P_{25360}-for	GCGGCCGCTGTATCCATATGAAGTAAGTGATAAGGTAGCT	forward primer used for CLJU_c25360 overexpression
G_{25360}-rev	ACGACGGCCAGTGCCAAGCTTTTATGCTGTAAATTTATTATTTTTTTGTG	reverse primer used for CLJU_c25360 overexpression
P_{20480}-for	GCGGCCGCTGTATCCATATGATGCATGATAAACAGAGTTCT	forward primer used for CLJU_c20480 overexpression
P_{20480}-rev	AATAATAACATCATACTTGCTCATTTTTAGACCTCCTTAAATCGGTAA	reverse primer used for CLJU_c20480 overexpression
G_{20480}-for	TTACCGATTTAAGGAGGTCTAAAAATGAGCAAGTATGATGTTATTATT	forward primer used for CLJU_c20480 overexpression
G_{20480}-rev	ACGACGGCCAGTGCCAAGCTTTTAGAGCTTAGGTGCTATTTTG	reverse primer used for CLJU_c20480 overexpression
P_{15270}-for	GCGGCCGCTGTATCCATATGCTTTATATCCTCCTTTTAATAAATTGT	forward primer used for CLJU_c15270 overexpression
G_{15270}-rev	ACGACGGCCAGTGCCAAGCTTTTATAAAATTACGTCTCTATTACAATCC	reverse primer used for CLJU_c15270overexpression
Universal-for	CCCCGTATCAAAATTTAGGAGGTTAGGATCCAATTTCTACTGTTGTAGAT	Universal forward primer of gene repression
Universal-rev	CCGTCGACCCCGGGCCATGGATAAAAATAAGAAGCCTGCAAATGCAGG	Universal reverse primer gene repression
gssR-crRNA-for	GGATCCAATTTCTACTGTTGTAGATGAAGACATTTGGAAAGAGTATCAA	forward primer used for *gssR* repression
gssR-crRNA-rev	TAAGAAGCCTGCAAATGCAGGCTTCTTATTTTTATTTGATACTCTTTCCAAATGTCTTC	reverse primer used for *gssR* repression
37350-crRNA-for	GGATCCAATTTCTACTGTTGTAGATAAATCAGGAAAGTACAACAATACA	forward primer used for CLJU_c37350 repression
37350-crRNA-rev	TAAGAAGCCTGCAAATGCAGGCTT	reverse primer used for

	CTTATTTTTATTGTATTGTTGTACTT TCCTGATTT	CLJU_c37350 repression
13550-crRNA-for	GGATCCAATTTCTACTGTTGTAGAT ATGAAGAGGTTAGAACTTATTATT	forward primer used for CLJU_c13550 repression
13550-crRNA-rev	TAAGAAGCCTGCAAATGCAGGCTT CTTATTTTTATAATAATAAGTTCTA ACCTCTTCAT	reverse primer used for CLJU_c13550 repression
15260-crRNA-for	GGATCCAATTTCTACTGTTGTAGAT TGCTCACCTGGGAAGCCTACAGAG	forward primer used for CLJU_c15260 repression
15260-crRNA-rev	TAAGAAGCCTGCAAATGCAGGCTT CTTATTTTTATCTCTGTAGGCTTCCC AGGTGAGCA	reverse primer used for CLJU_c15260 repression
21480-crRNA-for	GGATCCAATTTCTACTGTTGTAGAT TTAAATATGGAAGAGTTACATGAT	forward primer used for CLJU_c21480 repression
21480-crRNA-rev	TAAGAAGCCTGCAAATGCAGGCTT CTTATTTTTATATCATGTAACTCTTC CATATTTAA	reverse primer used for CLJU_c21480 repression
21500-crRNA-for	GGATCCAATTTCTACTGTTGTAGAT GTGTAGATCAAGTACTTGAATCCA	forward primer used for CLJU_c21500 repression
21500-crRNA-rev	TAAGAAGCCTGCAAATGCAGGCTT CTTATTTTTATTGGATTCAAGTACTT GATCTACAC	reverse primer used for CLJU_c21500 repression
P_{30000}-crRNA-for	GGATCCAATTTCTACTGTTGTAGAT ACTTTTAATAATATTATTCTAAAT	forward primer for CRISPRi plasmid repressing purine metabolism
P_{30000}-crRNA-rev	TAAGAAGCCTGCAAATGCAGGCTT CTTATTTTTATATTTAGAATAATATT ATTAAAAGT	reverse primer for CRISPRi plasmid repressing purine metabolism
P_{17370}-crRNA-for	AATTTCTACTGTTGTAGATTACTGG CTATAAATCCTACCCCAC	forward primer for CRISPRi plasmid repressing glutamate metabolism
P_{17370}-crRNA-rev	TAAGAAGCCTGCAAATGCAGGCTT CTTATTTTTATGTGGGGTAGGATTT ATAGCCAGTA	reverse primer for CRISPRi plasmid repressing glutamate metabolism
birA-ex-for	CAGTGGTGGTGGTGGTGGTGCTCG AGTTAAATATATTTATCTAAAC	forward primer used for BirA purification
birA-ex-rev	GCCTGGTGCCGCGCGGCAGCCATA TGATGAAACATTATGAAGTTC	reverse primer used for BirA purification
gssR-ex-for	GGTGGTGCCTCGAGTTAATTTTGTT CTCTAAAAGAATATAC	forward primer used for GssR purification
gssR-ex-rev	CTGGTGCCGCGCGGCAGCCATATG	reverse primer used for GssR

	ATGGAGAATGTTGACAAC	purification
CAC1850-381, 382s-IBS	GCTCGAGATAATTATCCTTAGTTTTCGATGACGTGCGCCCAGATAGGGTG	Targetron primer for CAC1850
EBS universal primer	CGAAATTAGAAACTTGCGTTCAGTAAAC	universal primer
CAC1850-381，382s-EBS2	TGAACGCAAGTTTCTAATTTCGATTAAAACTCGATAGAGGAAAGTGTCT	Targetron primer for CAC1850
CAC1850-381，382s-EBS1d	CAGATTGTACAAATGTGGTGATAACAGATAAGTCGATGACGTTAACTTACCTTTCTTTGT	Targetron primer for CAC1850
Cbei1890-357，358s-IBS primer	TGCTCGAGATAATTATCCTTAAACATCCGTGCAGTGCGCCCAGATAGGGTG	Targetron primer for Cbei1890
Cbei1890-357，358s-EBS2 primer	TGAACGCAAGTTTCTAATTTCGGTTATGTTCCGATAGAGGAAAGTGTCT	Targetron primer for Cbei1890
Cbei1890-357，358s-EBS1d primer	AGATTGTACAAATGTGGTGATAACAGATAAGTCCGTGCAACTAACTTACCTTTCTTTGT	Targetron primer for Cbei1890
qRT-16720-for	CAACAGGGAGATACAATACA	forward qRT-PCR primer for CLJU_c16720
qRT-16720-rev	TACCAATTTCGGTAACACTA	reverse qRT-PCR primer for CLJU_c16720
qRT-15260-for	ATACAAGCTGCATTATTCTC	forward qRT-PCR primer for CLJU_c15260
qRT-15260-rev	CATACATCACTTAGCAAGGA	reverse qRT-PCR primer for CLJU_c15260
qRT-21480-for	AAAGCCTTCCAAGTTATGAT	forward qRT-PCR primer for CLJU_c21480
qRT-21480-rev	GATTTGAACAGTCCAAGAGG	reverse qRT-PCR primer for CLJU_c21480
qRT-39590-for	AGTATTGTCATTTCAAGGTG	forward qRT-PCR primer for CLJU_c39590
qRT-39590-rev	AAAAGTTTCCCTATTGTGGT	reverse qRT-PCR primer for CLJU_c39590
qRT-16510-for	ACTTTCACAGGTGGTAATGG	forward qRT-PCR primer for CLJU_c16510
qRT-16510-rev	TTAATGTCGGCACTTTCATC	reverse qRT-PCR primer for CLJU_c16510
qRT-28160-for	GTTTCGTTGGTAGTTGGTGT	forward qRT-PCR primer for CLJU_c28160
qRT-28160-rev	GGTAGTGGCAATACGCTGTC	reverse qRT-PCR primer for

		CLJU_c28160
qRT-19400-for	ATAGAATTACACCGTTTAGG	forward qRT-PCR primer for CLJU_c19400
qRT-19400-rev	TCACAGTCATTGATGCAGGT	reverse qRT-PCR primer for CLJU_c19400
qRT-30460-for	ATAGAATTACACCGTTTAGG	forward qRT-PCR primer for CLJU_c30460
qRT-30460-rev	GATCTATATTCTTCAAAACCAACAGTATGATC	reverse qRT-PCR primer for CLJU_c30460
qRT-30450-for	GGTACAAATACCACAGAATGTCATCC	forward qRT-PCR primer for CLJU_c30450
qRT-30450-rev	CTTTGAAATCCTCAAAATTCTCAGTTC	reverse qRT-PCR primer for CLJU_c30450
qRT-29580-for	TCAGTAGTAGCGGTCATCTG	forward qRT-PCR primer for CLJU_c29580
qRT-29580-rev	TAAAAGCTTAGGCACTTTTC	reverse qRT-PCR primer for CLJU_c29580
qRT-37350-for	ATGTAACCCATTCGTTCACC	forward qRT-PCR primer for CLJU_c37350
qRT-37350-rev	ATGTAACCCATTCGTTCACC	reverse qRT-PCR primer for CLJU_c37350
qRT-21500-for	AAATCATCAAGGTGCCCATC	forward qRT-PCR primer for CLJU_c21500
qRT-21500-rev	ATGTAGCTGCATCTTCGTAT	reverse qRT-PCR primer for CLJU_c21500
qRT-30440-for	AGCATTGTTTTCGGGTATTG	forward qRT-PCR primer for CLJU_c30440
qRT-30440-rev	TTCCAGCTAAAAGGCCAACC	reverse qRT-PCR primer for CLJU_c30440
qRT-16690-for	TCCAAAATAGTAGAACCAAC	forward qRT-PCR primer for CLJU_c16690
qRT-16690-rev	CTTTAAAATCCTGAAGTGAT	reverse qRT-PCR primer for CLJU_c16690
qRT-13740-for	GATACAAAGAAAGCTGAACG	forward qRT-PCR primer for CLJU_c13740
qRT-13740-rev	GCTCCTTATTAATCCAATAT	reverse qRT-PCR primer for CLJU_c13740
qRT-28830-for	ACAATATCTCCAGGAACCCT	forward qRT-PCR primer for CLJU_c28830
qRT-28830-rev	GTTCATCTCCACCTTTCTCC	reverse qRT-PCR primer for CLJU_c28830
qRT-16780-for	TCAAGTGGATACTCAAAATG	forward qRT-PCR primer for

		CLJU_c16780
qRT-16780-rev	CACCTTCTTTAAAATCTGTG	reverse qRT-PCR primer for CLJU_c16780
qRT-13550-for	AAGACCTGCAACTCTATTAG	forward qRT-PCR primer for CLJU_c13550
qRT-13550-rev	TACTGCTAGTGATTCATCAT	reverse qRT-PCR primer for CLJU_c13550
qRT-00300-for	AAACATGGATGGATACGTTC	forward qRT-PCR primer for CLJU_c00300
qRT-00300-rev	CCAGTATCTTTTGCATTACC	reverse qRT-PCR primer for CLJU_c00300
qRT-05590-for	ATGAAATTTGGTAAGTTTAAAT	forward qRT-PCR primer for CLJU_c05590
qRT-05590-rev	TCAAACACACTTTTTAAAAAC	reverse qRT-PCR primer for CLJU_c05590
qRT-29900-for	ATGGAATAAAAGCTCTGC	forward qRT-PCR primer for CLJU_c29900
qRT-29900-rev	TGTCTCGTCCCAACTAAG	reverse qRT-PCR primer for CLJU_c29900
qRT-29910-for	GCTTGTAGATAAGGATGG	forward qRT-PCR primer for CLJU_c29910
qRT-29910-rev	AGTAAATGGCACCAGAAC	reverse qRT-PCR primer for CLJU_c29910
qRT-29920-for	TTGTAGTGGCTGGTGGTA	forward qRT-PCR primer for CLJU_c29920
qRT-29920-rev	TATAGGTACATGCTGCTC	reverse qRT-PCR primer for CLJU_c29920
qRT-29930-for	GTTAACTGGAGTAAAAGA	forward qRT-PCR primer for CLJU_c29930
qRT-29930-rev	TTTGTATTTCTCAACCCT	reverse qRT-PCR primer for CLJU_c29930
qRT-29940-for	TAGAGCAAGAGCTAAAAT	forward qRT-PCR primer for CLJU_c29940
qRT-29940-rev	AGGTCCTACAACGAGAAG	reverse qRT-PCR primer for CLJU_c29940
qRT-29950-for	AGTGGACATACCCTCCTT	forward qRT-PCR primer for CLJU_c29950
qRT-29950-rev	GAGGCAACTCCTTCAAAA	reverse qRT-PCR primer for CLJU_c29950
qRT-29960-for	ACAGCCATTCAGATGTAG	forward qRT-PCR primer for CLJU_c29960
qRT-29960-rev	AAGCCTCTTACTGTCTCC	reverse qRT-PCR primer for

		CLJU_c29960
qRT-29970-for	TTATGGGTGCGGTTATGG	forward qRT-PCR primer for CLJU_c29970
qRT-29970-rev	GTCCGAAACTGTTTGATTAGC	reverse qRT-PCR primer for CLJU_c29970
qRT-29980-for	ATAAATTCGGCTATACCAC	forward qRT-PCR primer for CLJU_c29980
qRT-29980-rev	AATGATGAGGATAGGAGC	reverse qRT-PCR primer for CLJU_c29980
qRT-29990-for	TGGTGACGTAGTTGCTAA	forward qRT-PCR primer for CLJU_c29990
qRT-29990-rev	TAAACCTAATTTCCTTGC	reverse qRT-PCR primer for CLJU_c29990
qRT-30000-for	TTAGGTGATGGTGGTTGG	forward qRT-PCR primer for CLJU_c30000
qRT-30000-rev	AGTCCATGTATCCGAAGC	reverse qRT-PCR primer for CLJU_c30000
qRT-17370-for	GAGGAAATAGAAACTGGATG	forward qRT-PCR primer for CLJU_c17370
qRT-17370-rev	CATAAGTGCATGTGGTAGGG	reverse qRT-PCR primer for CLJU_c17370
qRT-17380-for	CTCTGTGAAGGCTCTTGTAC	forward qRT-PCR primer for CLJU_c17380
qRT-17380-rev	AGATGGTCCTGAACCTACTA	reverse qRT-PCR primer for CLJU_c17380
qRT-28670-for	ATGCTCGAAGCAAGGTTA	forward qRT-PCR primer for CLJU_c28670
qRT-28670-rev	GTCCAGGATTTTCACTATTT	reverse qRT-PCR primer for CLJU_c28670
qRT-28690-for	GAGAAGGGCCATTTCCAG	forward qRT-PCR primer for CLJU_c28690
qRT-28690-rev	GAAGGCCACTTTGCATCA	reverse qRT-PCR primer for CLJU_c28690
qRT-28710-for	TTGCCGCACTTGGATTAG	forward qRT-PCR primer for CLJU_c28710
qRT-28710-rev	TAGCTGCACCACCGATTA	reverse qRT-PCR primer for CLJU_c28710
qRT-28720-for	TAGAGGAATGTGGAGCAG	forward qRT-PCR primer for CLJU_c28720
qRT-28720-rev	AACGTCCTACAGCAATAA	reverse qRT-PCR primer for CLJU_c28720
qRT-28730-for	GTTAAAGCTGCAAAAGAA	forward qRT-PCR primer for

		CLJU_c28730
qRT-28730-rev	TGCTCATAAGAACGATTA	reverse qRT-PCR primer for CLJU_c28730
qRT-28740-for	GCTAAGGGTGAAACAGGA	forward qRT-PCR primer for CLJU_c28740
qRT-28740-rev	CATTTGCACCGTATCTGT	reverse qRT-PCR primer for CLJU_c28740
qRT-28750-for	ACTTGACTATGCTTATCTT	forward qRT-PCR primer for CLJU_c28750
qRT-28750-rev	TTGTATTTACTGACCCAC	reverse qRT-PCR primer for CLJU_c28750
rho-for	AGATAGTGCAAGGGGAGTAC	forward qRT-PCR primer for *rho*
rho-rev	ACTTTACCGCAAACTTCATC	reverse qRT-PCR primer for *rho*
EMSA-37350-for	AGCCAGTGGCGATAAGGTTTTATTCACAGTGTGTATG	forward primer for EMSA
EMSA-37350-rev	AGCCAGTGGCGATAAGCAAGAGCCCCTATAATTACT	reverse primer for EMSA
EMSA-13550-for	AGCCAGTGGCGATAAGGCATTATAGTTTCTAAGCAAAG	forward primer for EMSA
EMSA-13550-rev	AGCCAGTGGCGATAAGGTAAAATTACAATTGTAATCGTAATG	reverse primer for EMSA
EMSA-08930-for	AGCCAGTGGCGATAAGTTTTACAGAAAAGAGCCACT	forward primer for EMSA
EMSA-08930-rev	AGCCAGTGGCGATAAGGCTCAGCTCTCTTAATTTTC	reverse primer for EMSA
EMSA-37390-for	AGCCAGTGGCGATAAGCGATATTGATTTTCTGTATAAAG	forward primer for EMSA
EMSA-37390-rev	AGCCAGTGGCGATAAGGTAATCCATCCCCTTTTATAA	reverse primer for EMSA
EMSA-30000-for	AGCCAGTGGCGATAAGAGCCAGTGGCGATAAGTATCAGTTCCCCCTTATTAT	forward primer for EMSA
EMSA-30000-rev	AGCCAGTGGCGATAAGCCAAAATCATAAAAATGTAT	reverse primer for EMSA
